# Selective Disruption of Salience‐Network Anterior Insula Connectivity in Misophonia: A Disorder‐Specific Neural Signature

**DOI:** 10.1002/hbm.70468

**Published:** 2026-02-12

**Authors:** Heather A. Hansen, Jordan E. Norris, Catherine M. Bain, Lauren E. Ethridge, Christine L. Tardif

**Affiliations:** ^1^ McConnell Brain Imaging Centre Montreal Neurological Institute Montreal Quebec Canada; ^2^ Department of Psychological Sciences William & Mary Williamsburg Virginia USA; ^3^ Department of Psychology University of Oklahoma Norman Oklahoma USA; ^4^ Department of Pediatrics Rush University Medical Center Chicago Illinois USA; ^5^ Department of Pediatrics, Section of Developmental and Behavioral Pediatrics University of Oklahoma Health Oklahoma City Oklahoma USA; ^6^ Department of Neurology & Neurosurgery McGill University Montreal Quebec Canada; ^7^ Department of Biomedical Engineering McGill University Montreal Quebec Canada

**Keywords:** anxiety, depression, insula, misophonia, resting‐state connectivity, salience network, seed‐based connectivity

## Abstract

Misophonia, a disorder characterized by extreme aversion to certain sounds, affects 5%–20% of the general population, yet mechanisms are still largely unknown. Recent neuroimaging studies have reported abnormal functional connectivity of the anterior insula to various limbic, salience, and motor regions in smaller samples of misophonic individuals versus controls, suggesting potential differences in underlying attentional or emotional processes. These findings prompt questions about the insular connectivity profile in larger samples of adults, what patterns emerge when the samples span a wider range of misophonia severity, and how these patterns may or may not overlap with other co‐occurring disorders. To address these questions, we analyzed resting‐state functional magnetic resonance imaging data from the open Welsh Advanced Neuroimaging Database (*N* = 162) comprising participants recruited from the general adult population and assessed for sensory sensitivity, anxiety, depression, and autistic traits. A misophonia severity score was derived from the sensory sensitivity data using a model trained on a second adult self‐report sample from Oklahoma (*N* = 777). Using anterior insula as a seed for a whole‐brain seed‐to‐voxel connectivity analysis, the derived misophonia severity scores were found to be significantly related to connectivity from the insula to clusters overlapping the planum temporale, operculum, precentral gyrus, and supplementary motor area. Notably, this insular connectivity profile was unique to the anterior insula of the salience network and was not observed when dividing the sample into misophonia (patient) versus control groups, or when grouping participants as a function of anxiety, depression, or autistic traits. These results underline the importance of the salience‐network anterior insula in understanding misophonic aversion and provide tentative evidence of neurological differences between misophonia and anxiety, depression, and autism. This work aids in our understanding of neural mechanisms of misophonia and emphasizes the benefit of treating misophonia as a continuous spectrum disorder to better reflect the variability of symptoms in the real world.

## Graphical abstract

Using resting‐state functional connectivity in a large sample of adults from the general population, our results provide neural evidence that misophonia is a discrete spectrum disorder (separate from anxiety, depression, and autism) and has a characteristic neural signature of salience insular connectivity.

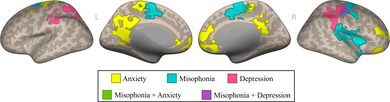

## 1 Introduction

While many people identify specific sounds as aversive, individuals with misophonia experience extreme reactions to everyday sounds (e.g., chewing gum, sniffling, clicking a pen), to an extent that can significantly impact their daily lives. Formally defined by a consensus committee in 2022, misophonia is a decreased tolerance to specific sounds (i.e., “triggers”) or associated stimuli, resulting in atypically strong negative emotional, physiological, and behavioral responses (Swedo et al. 2022). Population‐level estimates suggest that the prevalence of misophonia varies between 5% and 18% worldwide (Dixon et al. 2024; Vitoratou et al. 2023; Jakubovski et al. 2022; Kılıç et al. 2021), with upwards of 20% of surveyed college students experiencing significant symptoms (Wu et al. 2014; Zhou et al. 2017; Naylor et al. 2021). Misophonia experiences can range from mild to severe and present in a variety of ways (Swedo et al. 2022; Norris et al. 2022), leading researchers to suggest misophonia symptoms likely lie on a spectrum (Norris et al. 2022). Unfortunately, given the nascency of misophonia research, little is known about its neural etiology or mechanistic similarity to other mental health disorders.

Neuroimaging studies on misophonia have converged on a key region of importance: the anterior insula. The anterior insular cortex is implicated in a wide variety of functions potentially relevant for misophonia, including processing of disgust (Krolak‐Salmon et al. 2003), subjective evaluation of pain (Brooks et al. 2002), goal‐directed attentional control (Nelson et al. 2010; Eckert et al. 2009), and interoception (Wang et al. 2019). The first functional magnetic resonance imaging (fMRI) study of misophonia found that trigger sounds increased activation in the anterior insular cortex for individuals with misophonia (*N* = 20) as compared to age‐ and sex‐matched neurotypical controls (*N* = 22) (Kumar et al. 2017). A study shortly thereafter showed increased activation in the insula and in the anterior cingulate cortex in individuals with misophonia (*N* = 21) compared to controls (*N* = 23) when presented with audiovisual triggers (Schröder et al. 2019). Other recent work reported eight of their 11 misophonic participants showing activation to trigger sounds in at least one of the following regions: insula, cingulate cortex, hippocampus, or ventromedial prefrontal cortex (Grossini et al. 2022).

Additional insights into the misophonic experience have been gained via differences in functional connectivity, which measures how brain regions activate in synchrony. Specifically, stronger connectivity was seen in individuals with misophonia between the insula and the following brain regions: the posterior cingulate cortex/precuneus, ventromedial prefrontal cortex, hippocampus, and amygdala (Kumar et al. 2017); ventral premotor cortex (Kumar et al. 2021); and dorsal motor and somatosensory cortex (Hansen et al. 2022). However, these studies were done with relatively small sample sizes (misophonia *N*s = 7–20; control *N*s = 12–22). Notably, atypical anterior insula functional connectivity is also a feature of other psychiatric disorders, including anxiety (Baur et al. 2013; Simmons et al. 2011), depression (Horn et al. 2010; Kandilarova et al. 2018), and autism (Di Martino et al. 2009; Uddin and Menon 2009), highlighting the necessity of cross‐diagnostic neuroimaging studies to examine common neural mechanisms.

Over the past decade, the co‐occurrence of misophonia with symptomatically overlapping disorders has been investigated using self‐report surveys and clinical assessments. According to these behavioral results, 9%–57% of misophonic adults report a diagnosis or symptoms of anxiety (Rosenthal et al. 2022; Yektatalab et al. 2022; Claiborn et al. 2020; Vitoratou, Uglik‐Marucha, Hayes, Erfanian, et al. 2021; Vitoratou, Uglik‐Marucha, Hayes, and Gregory 2021), 7%–48% report depression (Yektatalab et al. 2022; Claiborn et al. 2020; Vitoratou, Uglik‐Marucha, Hayes, Erfanian, et al. 2021; Vitoratou, Uglik‐Marucha, Hayes, and Gregory 2021; Erfanian et al. 2019; Jager et al. 2020), and 3%–21% report autism spectrum disorder (Claiborn et al. 2020; Jager et al. 2020; Williams et al. 2022; Rinaldi et al. 2023). To put those results into context, prevalence of these disorders in the general population worldwide is reported to be around 7% for anxiety (Baxter et al. 2013), 11% for depression (Lim et al. 2018), and under 1% for autism (Zeidan et al. 2022), demonstrating elevated symptom presentation in misophonia. Similarly, studies have shown a positive correlation between misophonia severity and scores on assessments of anxiety (Wu et al. 2014; Zhou et al. 2017; Yektatalab et al. 2022; McKay et al. 2018; Wang et al. 2022; Guetta et al. 2022; Cassiello‐Robbins et al. 2021; Smit et al. 2023; Bagrowska et al. 2022; Siepsiak et al. 2020; Ay et al. 2024; Vitoratou et al. 2022; Cusack et al. 2018), depression (Wu et al. 2014; Zhou et al. 2017; Rosenthal et al. 2022; Yektatalab et al. 2022; Erfanian et al. 2019; McKay et al. 2018; Wang et al. 2022; Guetta et al. 2022; Cassiello‐Robbins et al. 2021; Smit et al. 2023; Siepsiak et al. 2020; Vitoratou et al. 2022; Remmert et al. 2022), and autism (Ertürk et al. 2024). However, as demonstrated by the wide co‐occurrence ranges observed in different studies, the current evidence for co‐occurrence between misophonia and other neuropsychiatric conditions is mixed: one group found no association between misophonia severity and anxiety or depression (Grossini et al. 2022), and another found a nominally significant negative association between rage‐related misophonia and autism (Smit et al. 2023). Thus, a deeper investigation incorporating neuroimaging is needed to assess co‐occurrence and overlapping neurocircuitry between misophonia and other neuropsychiatric conditions.

The present study seeks to add to our understanding of the etiology of misophonia through investigation of its neural profile and its relation to other disorders. Specifically, we seek to ascertain: (1) the anterior insula connectivity profile of misophonia and its relationship to symptom severity and (2) the extent to which this profile overlaps with that of anxiety, depression, and/or autism. We address these questions in a large sample (*N* = 162) from the general population, leveraging data from a new open‐access neuroimaging dataset (McNabb et al. 2025). We investigate misophonia as a spectrum disorder (i.e., with symptomology as a continuous measure), which better reflects the variability of misophonia symptoms in the real world (Norris et al. 2022). We present results from seed‐based connectivity analyses of the anterior insula covarying for misophonia symptom severity, investigating multiple subdivisions of the insula to probe the specificity of the insula in the neural profile of misophonia. We then repeat the analyses to assess relationships to symptoms of anxiety, depression, and autism in the same sample, teasing apart misophonia's neural signature from that of related and often co‐occurring conditions.

## 2 Method

### 2.1 Participants

#### 2.1.1 WAND Sample

The Welsh Advanced Neuroimaging Database (WAND) is a newly released open‐access, large‐scale, multimodal dataset (McNabb et al. 2025). The entire dataset comprises 178 adults enrolled across eight unimodal sessions of neuroimaging, cognitive tasks, and questionnaire assessments. Because the present study seeks to investigate functional connectivity in participants with questionnaire information, only participants who completed both the 3T functional MRI and trait questionnaires were included in analyses, resulting in 162 individuals. For a demographic breakdown of the WAND sample, see Table 1.

**TABLE 1:** Demographics of participants comprising the WAND and Oklahoma samples.

Demographic variable	WAND dataset (*N* = 162)		Oklahoma dataset (*N* = 777)	
	*N*	%	*N*	%
Sex/Gender^a^				
Male	58	35.8	186	23.9
Female	104	64.2	580	74.6
Nonbinary/prefer not to answer	—	—	11	1.4
Age				
Mean	30 years		19.4 years	
SD	10.9 years		3.3 years	
Range	18.3–63.7 years		18–63 years	
Count				
18–19.9 years	16	9.9	587	75.5
20–29.9 years	86	53.1	182	23.4
30–39.9 years	32	19.8	3	0.4
40–49.9 years	18	11.1	2	0.3
50+ years	10	6.2	3	0.4
Ethnicity^b^				
American Indian or Alaska Native^c^	—	—	19	2.4
Asian^d^	20	12.3	60	7.7
Black^e^	8	4.9	37	4.8
Hispanic or Latino^f^	—	—	85	10.9
White^g^	128	79.0	486	62.5
Mixed or Multiple ethnic groups^h^	4	2.5	84	10.8
Other ethnic group^i^	2	1.2	6	0.8

^a^ WAND collected sex (male, female), Oklahoma collected gender (*cis*/*trans* male, *cis*/*trans* female, nonbinary/prefer not to answer).

^b^ WAND categorized participant ethnicity based on classification from the 2021 UK census. Oklahoma gave participants ethnicity options to choose from.

^c^ Only Oklahoma provided American Indian or Alaska Native as an ethnicity option.

^d^ WAND reported as “Asian, Asian British or Asian Welsh”; Oklahoma reported as “Asian or Asian American.”

^e^ WAND reported as “Black, Black British, Black Welsh, Caribbean or African”; Oklahoma reported as “Black or African American.”

^f^ Only Oklahoma provided Hispanic or Latino as an ethnicity option.

^g^ WAND reported as “White”; Oklahoma reported as “White or Caucasian.”

^h^ WAND reported as “Mixed or Multiple ethnic groups”; Oklahoma allowed multiple answers to be selected.

^i^ WAND reported as “Other ethnic group”; Oklahoma reported “Other” and “Prefer not to answer.”

#### 2.1.2 Oklahoma Sample

Since the WAND dataset did not explicitly provide a misophonia questionnaire to participants, we sought to utilize an external dataset to construct and validate an indirect measure of misophonia. These data (hereafter “Oklahoma dataset”) were collected for other purposes at the University of Oklahoma and include 777 individuals recruited from the Psychology department research pool and the general university population via mass email. Briefly, participants completed an online survey containing a variety of misophonia, clinical, and demographic assessments. Responses to the individual assessment questions were not forced; handling of missing data is described in the relevant sections below. Data collection was approved by the University of Oklahoma Institutional Review Board (IRB#16450) and all individuals gave informed online consent to participate. In total, 1009 participants consented to the survey; 23% of respondents were removed for failing attention checks, providing the same answer for all questions, or for not completing the majority of questions. For a demographic breakdown of the Oklahoma dataset in comparison to WAND, see Table 1.

### 2.2 Behavioral Assessments

#### 2.2.1 Misophonia

All participants in the Oklahoma dataset completed the Misophonia Questionnaire (MQ) (Wu et al. 2014). The MQ has been used to assess misophonia in a wide variety of misophonia studies to date, including two influential neuroimaging projects (Kumar et al. 2017, 2021).

The MQ contains a Misophonia Symptoms Scale (seven items), a Misophonia Emotions and Behaviors Scale (10 items), and a Misophonia Severity Scale (one item). The Symptoms scale prompts “In comparison to other people, I am sensitive to the sound of …” followed by seven categories of common misophonia triggers (e.g., “People eating,” “Repetitive tapping”), with response options ranging from 0 (not at all true) to 4 (always true). The Emotions and Behaviors Scale prompts “Once you are aware of the sound(s), because of the sound(s), how often do you …” followed by 10 response behaviors (e.g., “Leave the environment,” “cover your ears”), with response options ranging from 0 (never) to 4 (always). These two scales sum to a maximum total MQ score of 68, with higher scores indicating more severe misophonia; prior work suggests a cutoff of 30 likely indicates the presence of misophonia (Möllmann et al. 2023). Lastly, the Severity Scale asks participants to self‐report the severity of their sound sensitivity on a labeled scale escalating from 1 (minimal) to 15 (very severe) or check a box indicating they do not have any sound sensitivities, coded as 0. Scores seven or greater are suggested to indicate “clinical” misophonia (Wu et al. 2014; Möllmann et al. 2023).

Results showed an extremely high response rate for MQ items, with 100% of participants (*N* = 777) responding to each of the 17 subscale items and 99.9% (*N* = 776) responding to the single severity item. As only one participant did not respond to the severity question, we elected to not impute or correct for the missing case. Participants ranged in their total MQ scores between zero and 63 (mean = 24.4), with 240 (31%) scoring above the cutoff of 30. Self‐reported severity ranged from zero to 15 (mean = 4.4), with 147 (19%) falling above the cutoff of 7, consistent with reported prevalence in other college samples (Zhou et al. 2017; Naylor et al. 2021; Norris et al. 2022). For depictions of MQ analyses in the Oklahoma sample, see Figure S1.

#### 2.2.2 Sensory Sensitivity

All participants in both the WAND and the Oklahoma datasets completed the Adolescent/Adult Sensory Profile (AASP) (Brown et al. 2001). The AASP contains 60 statements about experiences across the senses (e.g., “I stay away from noisy settings,” “I add spice to my food”), with response options ranging from 1 (nearly never) to 5 (almost always). The statements split equally into four subscales (sensation avoiding, sensory sensitivity, sensation seeking, low registration), as per Dunn's (1997) model of the sensory profile (Dunn 1997).

Assessments in the WAND dataset were completed in full by all respondents. In the Oklahoma dataset, participants had an additional response option (“Not applicable to me”) for each question as well as the option to leave the question blank, both coded as 0. These two options accounted for 1.69% of the overall data.

#### 2.2.3 Anxiety, Depression

Participants in the WAND sample completed the Hospital Anxiety and Depression Scale (HADs) (Zigmond and Snaith 1983). The HADs is a 14‐item questionnaire probing the frequency or severity of symptoms with seven intermixed statements per condition (e.g., A: “I feel tense or ‘wound up’ …”; D: “I still enjoy the things I used to enjoy …”). Each statement is accompanied by varied text anchors on a scale from 0 to 3, with higher scores indicating more presence of the condition. Scores range from 0 to 21 for each condition, with scores of 11 or more indicating “definite” clinical cases (Zigmond and Snaith 1983).

#### 2.2.4 Autism

Autistic traits in the WAND sample were assessed using the abridged version of the adult Autism‐Spectrum Quotient (AQ‐S) (Hoekstra et al. 2011). The AQ‐S is a 28‐item questionnaire with intermixed statements probing difficulties with social skills, a preference for routine, attention‐switching difficulties, difficulties in imagination, and a fascination with numbers/patterns. Each statement is accompanied by a 4‐point Likert scale from “Definitely agree” to “Definitely disagree,” with points assigned per response depending on if the statement is indicative of autism (e.g., “I frequently get strongly absorbed into one thing”) or not (e.g., “I find social situations easy”). Scores range from 28 to 112, with a suggested cutoff of 65 or higher to distinguish individuals with autism from controls (Hoekstra et al. 2011).

#### 2.2.5 Deriving a Misophonia Score

To get an indirect measure of misophonia in the WAND dataset, we built a general linear regression model in the Oklahoma dataset that could predict misophonia (MQ total score) from one's sensory profile (AASP); the model was then applied to the AASP data from the participants in the WAND dataset. First, we randomly partitioned the 777 participants in the Oklahoma dataset into a training set (*N* = 615) and a test set (*N* = 162), constrained such that each set had an equal proportion of participants with and without misophonia (as defined by ≥ 7 and < 7 on the MQ Severity Scale, respectively). The participant with missing data for the MQ Severity Scale item was randomly assigned. The set sizes were chosen to equate the size of the test set in the Oklahoma sample with the size of the WAND sample. The training and test sets did not significantly differ in age (*t*(775) = −1.02, *p* = 0.307) or gender breakdown (*t*(775) = −1.85, *p* = 0.065).

Next, we built a model in the training set using the 30 AASP questions categorized as representing sensation avoiding and sensory sensitivity. This choice was made to reduce the number of predictors in the model (to avoid overfitting), and because these subscales had the highest correlations with MQ total scores (*r =* 0.42, *p* < 0.001 and *r =* 0.51, *p* < 0.001, respectively) in the entire Oklahoma sample. Responses to these 30 items and MQ total scores were standardized using *z* scores then input into the Matlab function *fitlm*. The resultant linear regression model was applied to the AASP data of the test set using *predict*. Model accuracy was determined by correlating the test participants' predicted MQ total scores with their actual MQ total scores and computing adjusted *R* ^2^. Additionally, we used a resampling approximation permutation test to further probe the significance of results (see Berry et al. 2011 for a review). A null distribution was created by randomly shuffling the MQ total scores of the test set 1000 times and calculating the resultant correlations for each permutation.

### 2.3 Neuroimaging Protocol and Analyses

#### 2.3.1 Acquisition

The neuroimaging data used in the present analyses derive from Session 3 of the WAND protocol (McNabb et al. 2025). Specifically, we utilize the single run of resting state blood oxygen level dependent (BOLD) fMRI data acquired at 3T. The resting state scan was acquired while participants looked at a white fixation cross presented on a black background for 10 min 38 s. Data were acquired with a multiband gradient echo planar imaging sequence (TR = 2000 ms, TE = 30 ms, voxel size = 2 × 2 × 2 mm^3^, flip angle = 70°). Additionally, a 1‐mm isotropic magnetization‐prepared rapid acquisition with gradient echo (MPRAGE) T1‐weighted anatomical scan was acquired on all participants.

#### 2.3.2 Preprocessing and Denoising

Resting‐state preprocessing was performed using CONN release 22.v2407 (Whitfield‐Gabrieli and Nieto‐Castanon 2012; Nieto‐Castanon and Whitfield‐Gabrieli 2022) and SPM 12 (Penny et al. 2011) release 12.7771. Data were preprocessed via the standard CONN pipeline using default parameters for seed‐to‐voxel connectivity analysis (see Nieto‐Castanon 2020). First, data were corrected for motion and magnetic susceptibility distortions using b‐spline interpolation in SPM (Andersson et al. 2001). Outliers were detected using Artifact Detection Tools (ART; Sladky et al. 2011) and defined as any time points in which framewise displacement was above 0.9 mm or the BOLD signal exceeded five standard deviations from the participant's global signal (Power et al. 2014; Nieto‐Castanon 2025); these timepoints were subsequently removed. Data were then normalized into MNI‐space, resampled to 2 mm isotropic voxels, and segmented by tissue class (gray matter, white matter, CSF). Finally, data were smoothed with a Gaussian kernel of 8 mm full width half maximum.

Data were denoised using the CONN toolbox standard parameters (Nieto‐Castanon 2020). Briefly, denoising included the nuisance regression of potential confounding effects characterized by white matter and CSF timeseries (five CompCor noise components each), outlier scans, motion parameters and their first order derivatives (12 noise terms), session and task effects and their first order derivatives (two noise terms), and linear trends within each functional run (two noise terms). Noise components for white matter and CSF timeseries were estimated using CompCor (Behzadi et al. 2007; Chai et al. 2012), which computed the average BOLD signal within the timeseries in addition to the largest principal components orthogonal to the average BOLD signal, motion parameters, and outlier scans within each participant's segmentation masks. The frequency of the BOLD timeseries was then bandpass filtered between 0.008 and 0.09 Hz. The effective degrees of freedom of the BOLD signal after denoising were estimated to range from 59.4 to 89.9 (average 86) across all participants.

Quality control of the data was performed by assessing mean motion (average 0.13 mm framewise displacement), proportion of valid scans after outlier removal (average 0.96), and functional and structural normalization alignment. Of the 162 WAND participants, nine were excluded from the functional connectivity analyses due to being deemed an extreme outlier by CONN for one or more quality assurance measures (more than 3 times the interquartile range past the third quartile). Specifically, two participants were removed for mean motion (> 0.34 mm), six were removed for proportion of valid scans (< 0.8), and one was removed for poor functional normalization. Thus, 153 participants comprise the analyses described here.

#### 2.3.3 Functional Connectivity

First‐ and group‐level analyses were performed using the CONN toolbox. Seed‐based connectivity maps were estimated within each participant, using the bilateral anterior insula as seeds unless otherwise noted, defined by the default CONN network parcellation atlas (networks.Salience.AInsula, L: −44,13,1; R: 47,14,0). Functional connectivity strength was represented by Fisher‐transformed bivariate correlation coefficients from a weighted general linear model (GLM; Nieto‐Castanon 2020), estimated separately for each seed area and target voxel, modeling the association between their BOLD signal timeseries and convolved with an SPM canonical hemodynamic response function.

For each individual voxel a separate GLM (Nieto‐Castanon 2020) was estimated, with first‐level connectivity measures at this voxel as dependent variables and subject‐level identifiers as independent variables. Voxel‐level hypotheses were evaluated using multivariate parametric statistics with random effects across participants and sample covariance estimation across multiple measurements. Inferences were performed at the level of individual clusters (groups of contiguous voxels). Cluster‐level inferences were based on parametric statistics from Gaussian Random Field theory (Nieto‐Castanon 2020; Worsley et al. 1996). Results were thresholded using a combination of a cluster‐forming *p* < 0.01 voxel‐level threshold and a familywise error corrected *p*‐FWE < 0.025 one‐sided cluster‐size threshold (Chumbley et al. 2010), unless otherwise stated.

Labeling of clusters was performed in BSPMview (v.20180918) using the Harvard–Oxford cortical atlas (Desikan et al. 2006), with cluster peaks separated by 6 mm and redundant labels excluded. Note that this atlas was used to aid in descriptive interpretation, not as an analytical tool.

## 3 Results

### 3.1 Sensory Behaviors Can Serve as a Viable Proxy for Misophonia Severity

#### 3.1.1 Prediction Accuracy of Model in Oklahoma Sample

**FIGURE 1:**
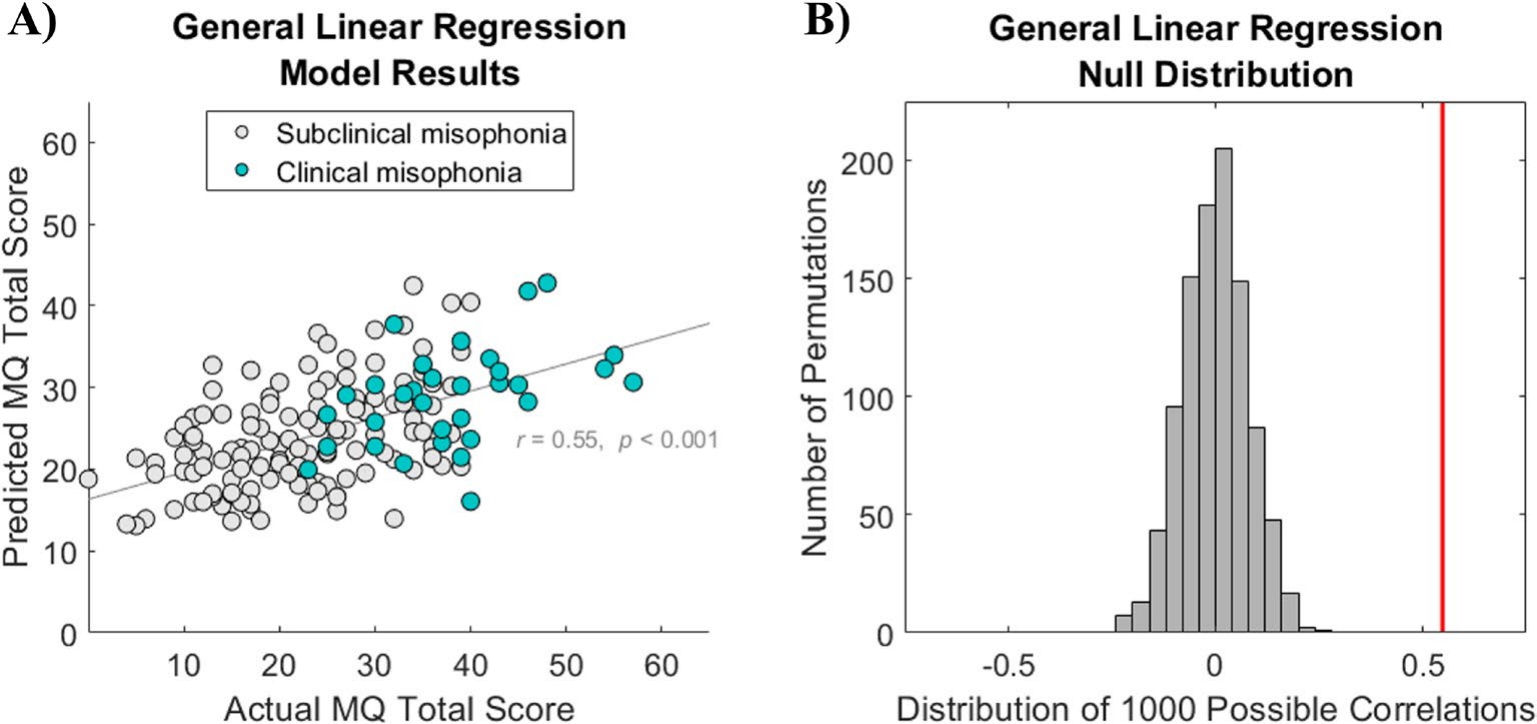
General linear regression model results. (A) Actual MQ scores versus MQ scores predicted by the model. Teal circles denote individuals who self‐reported ≥ 7 (out of 15) on the MQ Severity Scale, indicating clinical levels of misophonia, as is common in the field. Gray circles denote individuals who self‐reported < 7. The participant with missing data for the MQ severity item is not visualized on the plot but is otherwise included in all analyses of MQ total score. (B) Null distribution of 1000 possible correlations attainable by shuffling predicted scores. The red line denotes the actual correlation observed (*r* = 0.55).

The model results from the Oklahoma test set are depicted in Figure 1. MQ total scores predicted by the model correlated with actual MQ total scores at *r =* 0.55, *p* < 0.001 (Figure 1A). Adjusted *R* ^2^ for the model was 0.326, suggesting the 30 AASP questions accounted for 32.6% of the variance in MQ total score after accounting for the number of predictors. Classification of the Oklahoma test set into individuals with misophonia (MQ total ≥ 30) versus controls (MQ < 30) was accomplished by the model with 0.48 sensitivity and 0.90 specificity (AUC = 0.796) (Zhang and Mueller 2005), with a positive predictive value of 0.74 and a negative predictive value of 0.75. A McNemar test comparing the classifier to a majority‐class (null) model showed that the classifier made significantly fewer errors than the null model, *χ* ^2^(1) = 8.31, *p* = 0.004. Finally, compared to the null distribution (Figure 1B), our observed correlation was higher than each of the random permutations and thus represented a significant model prediction (*p* < 0.001).

Probing the model more deeply, we investigated which predictors significantly impacted the model by analyzing the *p*‐values of the beta coefficients, using a Bonferroni correction to account for multiple comparisons. Three significant predictors were found (see Table 2): (1) relocating when smelling strong odors, (2) limiting distractions while working, and (3) being distracted if there is a lot of noise around. While being distracted by noise feels intuitive as a predictor of misophonia, aversion to strong odors is a bit more surprising. This item perhaps is reflective of individuals with misophonia experiencing heightened sensory sensitivity in general (Rinaldi et al. 2023; Andermane et al. 2023); we posit odor may have specifically been an informative predictor given the anterior insula's known involvement in olfaction and processing feelings of disgust (Roy‐Côté et al. 2021).

**TABLE 2:** Significant model coefficients in predicting MQ total score from AASP responses.

AASP item	Beta estimate	Beta SE	*t*‐Statistic	*p*
I leave or move to another section when I smell a strong odor in a store (e.g., bath products, candles, perfumes)	0.140	0.038	3.705	2.3 × 10^−4^
I limit distractions when I am working (e.g., I close the door, or turn off the TV)	−0.130	0.039	−3.385	7.6 × 10^−4^
I am distracted if there is a lot of noise around	0.145	0.045	3.211	1.4 × 10^−3^

*Note: p*‐Values reported for significant coefficients after a Bonferroni correction, i.e., *p* < 1.7 × 10^−3^ (0.05/30).

Abbreviations: AASP, Adolescent/Adult Sensory Profile; MQ, Misophonia Questionnaire; SE, standard error of the coefficients.

#### 3.1.2 Application of Model to WAND

**FIGURE 2:**
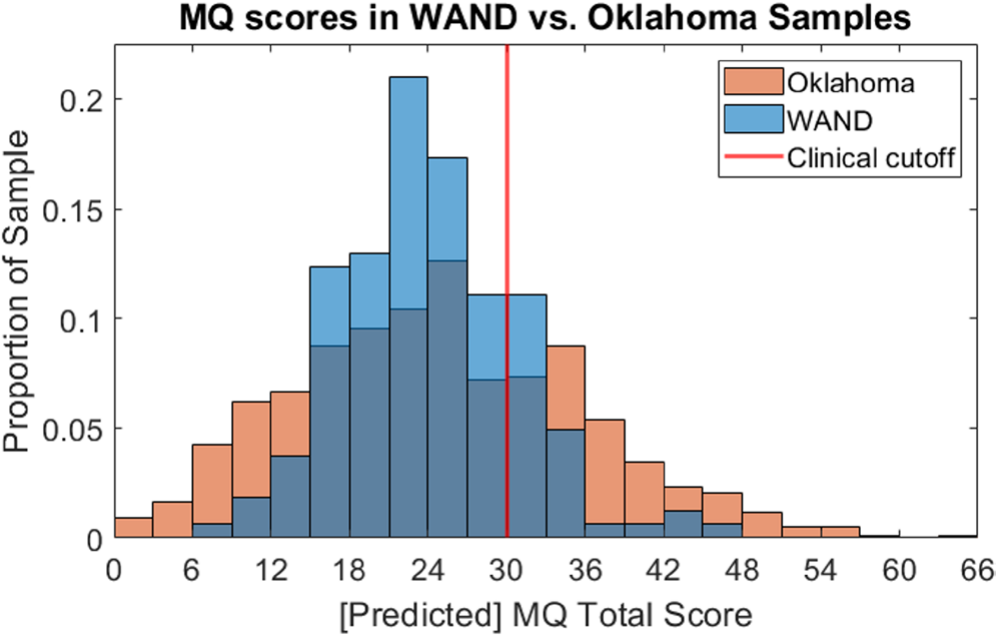
Histogram of predicted MQ scores in the WAND dataset, compared to MQ scores of Oklahoma dataset. *Y*‐axis is scaled by proportion of sample to aid in interpretability, since the sample sizes differed. The red line denotes the recommended clinical cutoff (MQ total score ≥ 30).

With successful construction of a model to predict misophonia (as defined by MQ total score) in the Oklahoma dataset, we applied this model to the AASP responses in the WAND dataset. Predicted MQ scores are depicted in Figure 2.

In the WAND dataset, participants ranged in their predicted MQ scores between 8.9 and 45.1 (mean = 24.2), with 31 (19%) scoring above the cutoff of 30 (Figure 2). This result demonstrates that (1) misophonia naturally occurs in a general population of neurotypical adults, and investigations into misophonia can occur in samples not specifically collected for the purpose and (2) the percentage of individuals with a clinical definition of misophonia in this WAND sample matches prior work on the prevalence of misophonia, specifically in a UK population (Vitoratou et al. 2023).

A comparison of the distributions of predicted MQ scores in the WAND dataset versus the Oklahoma dataset is depicted in Figure 2. Unsurprisingly, the two samples align in their mean MQ scores; while the intercept term of the model was not significant, the sample mean is generally a good baseline for a model prediction. The range of the MQ scores in the WAND sample is more restricted than in the Oklahoma sample, suggesting that the sensory profile questionnaire may not completely capture misophonia experiences at the extremes (i.e., absent or severe self‐reported misophonia symptoms). Although imperfect, these predicted MQ scores in the WAND dataset offer a useful proxy, allowing us to investigate misophonia in a dataset which does not otherwise explicitly probe misophonia.

### 3.2 Misophonia Has a Distinct Insular Connectivity Profile

With a sufficient estimate of misophonia severity in the WAND sample, we now turn to the first of our neuroimaging questions: what does insular connectivity look like in a large sample of individuals from the general population, and how does it change as a function of misophonia severity?

#### 3.2.1 Anterior Insula Connectivity Changes as a Function of Misophonia Severity, but Not Group Membership

**FIGURE 3:**
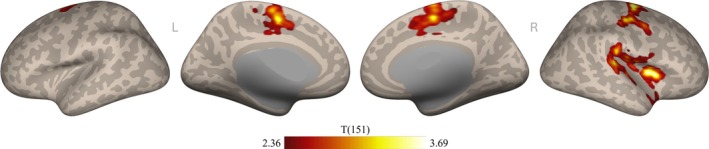
Seed‐based connectivity from the anterior insula to the rest of the brain as a function of misophonia severity. Connectivity maps are projected to the surface in MNI‐space for visualization. Significance thresholds depicted for *t*(151) > 2.36, using a voxel threshold of *p* < 0.01 and cluster threshold of *p* < 0.025 FWE corrected (one‐tailed, positive).

To assess the relationship between anterior insula connectivity and misophonia severity, we first performed a seed‐to‐voxel connectivity analysis from the bilateral anterior insula to every voxel in the brain, using predicted MQ total score as a covariate of interest. Results showed two clusters significantly positively correlated with the insula as a function of misophonia severity (Figure 3). No clusters were significantly negatively correlated with the insula as a function of misophonia severity. As illustrated by Figure 3, one of the significant clusters extended through five main regions (insular cortex, central opercular cortex, planum temporale, precentral gyrus, and temporal pole) and the other significant cluster extended through four main regions (precentral gyrus, supplementary motor cortex, superior frontal gyrus, and posterior cingulate gyrus). Note that since the anterior insula was used as a seed, significant voxels within the insular cortex may be explained by either posterior insula or contralateral anterior insula connectivity. Details on the list of regions comprising each cluster can be found in Table S1.

Additionally, for consistency with prior misophonia work that analyzed neural differences in patients versus controls (e.g., Kumar et al. 2017; Schröder et al. 2019; Grossini et al. 2022; Kumar et al. 2021; Hansen et al. 2022), and to explore whether using misophonia as a continuous measure rather than a binary categorization altered results, we repeated the seed‐to‐voxel connectivity analysis with binarized group membership. A separate variable was constructed by labeling participants with predicted MQ total scores ≥ 30 as “patients” (*N* = 29) and MQ total scores < 30 as “controls” (*N* = 124) (Möllmann et al. 2023). Note that MQ total scores were used to binarize groups instead of the more common Severity Scale item of the MQ; as we derived MQ scores, the wider variability of MQ total scores in the Oklahoma dataset better supported prediction models than the more truncated Severity Scale item did. The resulting binarized group membership was used as a covariate of interest in the analysis instead of MQ total score. Notably, at the same voxel‐ and cluster‐thresholds as above, no significant clusters were observed. Thresholds were lowered to investigate this null result, revealing clusters aligning with Figure 3 only when thresholded very liberally (voxel‐level threshold *p* < 0.1, cluster‐size threshold *p*‐FWE < 0.1).

#### 3.2.2 The Misophonia Connectivity Profile Is Specific to the Anterior Insula of the Salience Network

**FIGURE 4:**
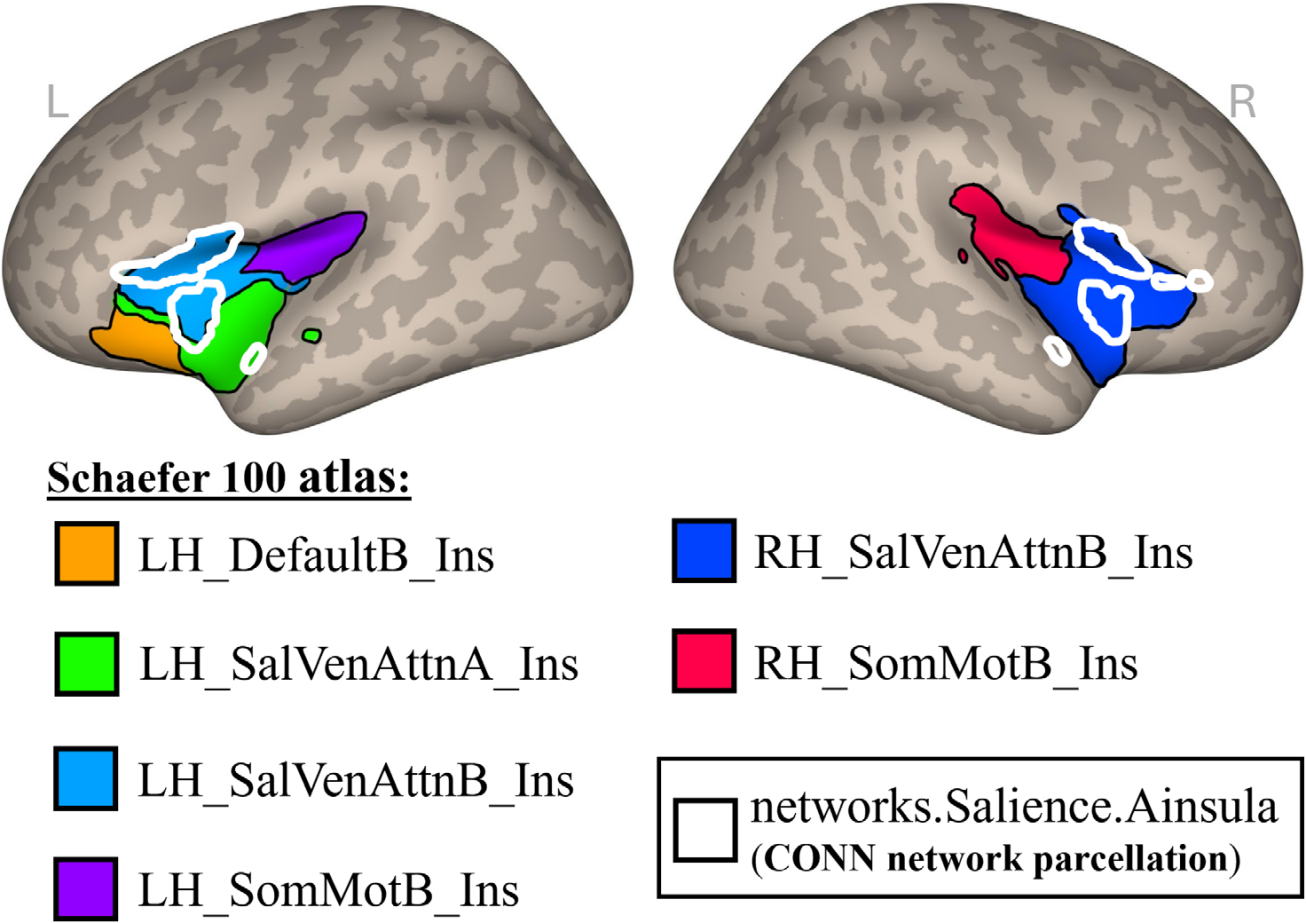
Location of insular subdivisions. Regions are projected to the surface in MNI‐space for visualization. The subdivisions in solid color are derived from the Schaefer 100 atlas (100‐parcel, 17‐network parcellation). The areas outlined in white denote the anterior insula seed provided by the CONN network parcellation. Ainsula, anterior insula; Default = default mode network; Ins, insula; LH, left hemisphere; RH, right hemisphere; SalVenAttn, salience/ventral‐attention network; SomMot, somatomotor network.

The prior analyses make use of the anterior insula seed, attributed to the salience network, in the default network parcellation provided by CONN. However, the insula can be subdivided in multiple ways and participates in more than just the salience network. To explore the specificity of the anterior insula in its relationship to misophonia, we switched to a larger atlas—Schaefer and colleagues' 100‐parcel 17‐network parcellation (Schaefer et al. 2018; Kong et al. 2021) (hereafter “Schaefer 100 atlas”)—with more insular granularity. We again repeated the seed‐to‐voxel connectivity analysis as a function of misophonia severity represented in Figure 3, but this time systematically varied the seed in separate analyses. In total, we analyzed all six insular subdivisions provided by the Schaefer 100 atlas (Schaefer et al. 2018; Kong et al. 2021), depicted in Figure 4: salience/ventral attention insular regions (LH_SalVenAttnA_Ins, LH_SalVenAttB_Ins, RH_SalVenAttnB_Ins), default mode insular region (LH_DefaultB_Ins), and somatomotor insular regions (LH_SomMotB_Ins, RH_SomMotB_Ins).

**FIGURE 5:**
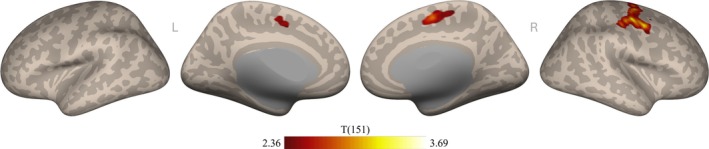
Seed‐based connectivity from the left hemisphere salience/ventral attention insula to the rest of the brain as a function of misophonia severity. Connectivity maps are projected to the surface in MNI‐space for visualization. Significance thresholds depicted for *t*(151) > 2.36, using a voxel threshold of *p* < 0.01 and cluster threshold of *p* < 0.025 FWE corrected (one‐tailed, positive).

First, we performed a seed‐to‐voxel connectivity analysis from the left‐hemisphere salience/ventral attention insular regions to every voxel in the brain, using predicted MQ total score as a covariate of interest. Results showed one cluster significantly positively correlated with this insula seed as a function of misophonia severity (Figure 5), extending mainly through the precentral gyrus, postcentral gyrus, and supplementary motor cortex. Details on the cluster can be found in Table S2. We note that a significant cluster was only found when averaging across both the SalVenAttnA and SalVenAttnB regions together and not when analyzed separately.

Next, we repeated the seed‐to‐voxel connectivity analysis from the right‐hemisphere salience/ventral attention insular region. No significant clusters were found to vary in connectivity as a function of misophonia severity. We likewise observed no significant clusters when seeding any of the other insular subdivisions, either separately or collapsed across hemispheres.

To confirm whether anterior insula connectivity is specific to a left hemisphere seed, we returned to the CONN parcellation to further tease apart the findings from Figure 3. We repeated the seed‐to‐voxel connectivity analysis with the left and right anterior insula parcels seeded separately, instead of averaging across them bilaterally. Significant clusters were found from both the left and right insula that were positively correlated with misophonia severity, the details of which are presented in Table S3. Since the combination of these clusters resembles the output of the analysis using the bilateral insula, we focus on the bilateral insula in our remaining analyses for simplicity.

### 3.3 The Connectivity Profile of Misophonia Distinguishes It From Putatively Related Disorders

In the previous analysis, we found a connectivity profile from the left salience‐network anterior insula that was significantly associated with misophonia severity. Given the WAND participants completed a plethora of questionnaires in addition to the AASP, from which we derived our measure of misophonia, we additionally utilized these other clinical metrics available in the WAND dataset. Specifically, we assessed how disorders with higher prevalence rates in individuals with misophonia (depression, autism, and anxiety) corresponded with misophonia in behavior or connectivity profile.

#### 3.3.1 Behavioral Results

**FIGURE 6:**
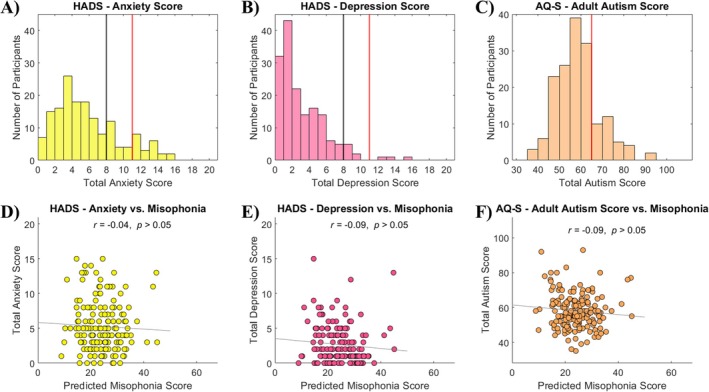
Behavioral results from clinical assessments. (A–C) Histograms of score distributions for anxiety (A), depression (B), and autistic traits (C). The red lines denote the recommended “clinical” cutoffs for each measure, and the black lines denote a “subclinical” cutoff. (D–F) Correlations between assessment scores and misophonia severity (predicted MQ score) for anxiety (D), depression (E), and autistic traits (F). Note that Autistic traits (C and F) represent a normed composite score from the AQ‐S, which lacks questions probing sensory dysfunction. AQ‐S, Autism‐Spectrum Quotient Short; HADS, Hospital Anxiety and Depression Scale.

In the WAND dataset, scores on the HADs revealed distributions of mild to moderate anxiety (*M* = 5.27, SD = 3.67) and mild depression (*M* = 2.67, SD = 2.72) (Figure 6). Per guidelines from Zigmond and Snaith (1983), 21 individuals (13%) would be considered clinical cases of anxiety, and 20 individuals (12%) would be considered borderline cases of anxiety (Figure 6A). On the depression subscale, three individuals (2%) would be considered clinical cases of depression, and seven individuals (4%) would be considered borderline cases of depression (Figure 6B). Scores on the AQ‐S revealed a comparable distribution (*M* = 58.07, SD = 10.10) to that presented by Hoekstra et al. (2011). Using their recommended cutoff, 30 individuals (19%) could be considered to have autistic traits (Figure 6C). Of note, co‐occurrence of anxiety, depression, or autistic traits did not markedly differ in the subgroup of misophonia “patients” compared to “controls” (see Table S4).

To put these co‐occurrence rates into context, we compared them to the Oklahoma dataset, in which individuals were asked to check a box signifying the presence or absence of a particular diagnosis. Based on this self‐report question, 23% of individuals reported Anxiety disorders, 20% of individuals reported Depression, and 0.6% of individuals reported Autism.

To ascertain any behavioral relationship with misophonia, scores from each assessment were correlated with predicted MQ total scores. None of the clinical measures estimated here (anxiety, depression, or autism) were significantly correlated with misophonia in this sample (Figure 6D–F). For interpretation of these findings, see Section 4. For comparison, MQ total scores in the Oklahoma Dataset showed small but significant point‐biserial correlations (Nagel 2025) with anxiety (*r*_pb_ *=* 0.15, *p* < 0.001), depression (*r*_pb_ *=* 0.13, *p* < 0.001), and autism (*r*_pb_ *=* 0.16, *p* < 0.001) when the disorders were represented as binary diagnoses.

#### 3.3.2 Connectivity Results

**FIGURE 7:**
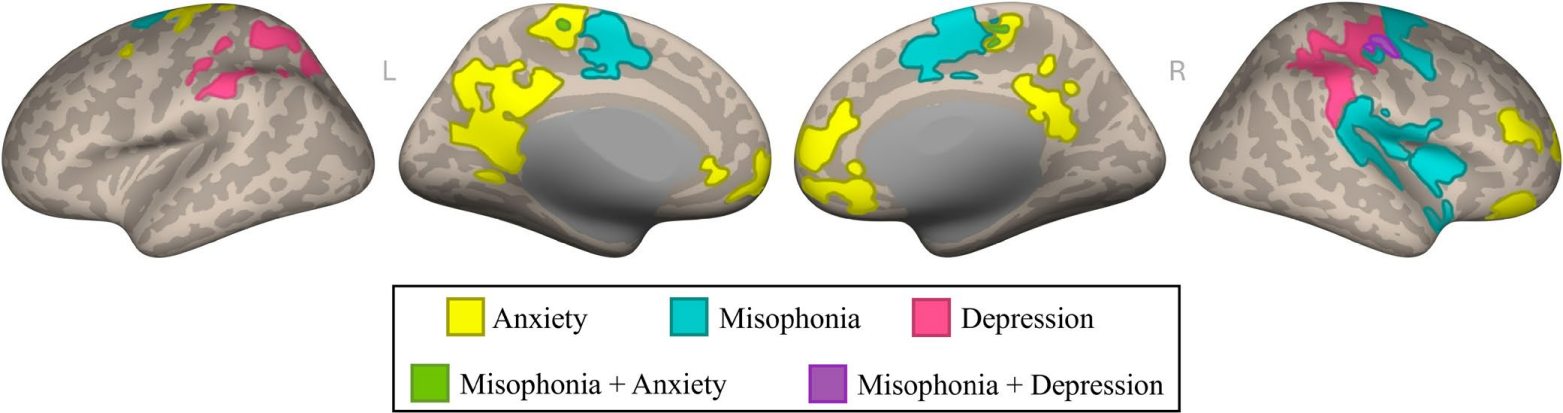
Connectivity profile of misophonia versus related clinical measures. Binarized depiction of insular connectivity maps associated with misophonia (teal), anxiety (yellow), and depression (pink), as well as regions of overlap (green, purple). Connectivity maps are projected to the surface in MNI‐space for visualization.

To assess the basic relationship between anterior insula connectivity and each clinical measure (anxiety, depression, and autism), we first performed seed‐to‐voxel connectivity analyses from the bilateral anterior insula to every voxel in the brain, treating each measure as a covariate of interest in a separate analysis. Results showed clusters significantly positively correlated with the insula as a function of both anxiety and depression (Figure 7). Notably, the observed clusters did not extend over regions found in the analysis of predicted MQ total score from Aim 2. No clusters were found to be significantly correlated with the insula as a function of autism.

While the observed clusters from the individual models provide useful starting points for how connectivity patterns relate to anxiety, depression, and autistic traits separately, they do not provide insight into whether those connectivity patterns represent unique or shared variance between measures. To explore whether the functional connectivity patterns observed in Figure 3 were unique to misophonia, we performed one final seed‐to‐voxel connectivity analysis from the bilateral anterior insula to every voxel in the brain, using misophonia level as a covariate of interest and controlling for all clinical measures (anxiety, depression, and autism) and demographic variables (age and sex) for which we had data.

Results showed that both clusters observed from the individual misophonia model in Figure 3 remained significantly related to misophonia level after controlling for covariates. As listed in Table S5, peak voxels in the first cluster fell within five main regions (insular cortex, central opercular cortex, planum temporale, parietal operculum cortex, and precentral gyrus), and peak voxels in the second cluster fell within five main regions (precentral gyrus, supplementary motor cortex, postcentral gyrus, superior frontal gyrus, and posterior cingulate gyrus). Importantly, although we observed small clusters of overlap from the individual models of misophonia, anxiety, and depression (green and purple in Figure 7), the clusters remain significantly associated with misophonia in the aggregate model when controlling for anxiety and depression.

## 4 Discussion

This study investigates the neural connectivity profile of misophonia in a large sample from the general population and explores potential overlapping etiology with anxiety, depression, and autism. Per previous research demonstrating the anterior insula as a region of interest in misophonia, we used a seed‐based connectivity approach with the bilateral anterior insula as seeds. The results from our analyses provide evidence of (1) an association between misophonia symptom severity and anterior insula connectivity strength, specifically in affiliation with the salience network and (2) independence of the insular connectivity profile of misophonia from anxiety, depression, or autistic traits.

### 4.1 Misophonia Symptom Severity Is Associated With Anterior Insula Connectivity Strength, Specifically in Affiliation With the Salience Network

First, we analyzed the connectivity profile of misophonia as a function of severity and found that the bilateral anterior insula connected to two clusters with significant association to MQ total scores. As self‐reported misophonic symptom severity increased, functional connectivity between the anterior insula and these clusters increased; this result supports previous research in highlighting the anterior insula as a key region relevant to misophonia. To probe the specificity of this finding, which used the bilateral anterior insula provided in the CONN toolbox belonging to the salience network, we sought alternate definitions of the anterior insula. We expanded to the Schaefer 100 atlas (Schaefer et al. 2018; Kong et al. 2021) and repeated the seed‐based connectivity analysis with insular subregions belonging to the salience/ventral attention network, the default mode network, and the somatomotor network. We observed significant connectivity as a function of misophonia only with the insula of the salience/ventral attention network, revealing a cluster that overlapped with the results from the CONN toolbox's anterior insula of the salience network. Thus, while the insula serves many roles in brain function, only insula connectivity associated with determining salience and attending to stimuli seems to be affected in misophonia. Kumar and colleagues (Kumar et al. 2017) and Schröder and colleagues (Schröder et al. 2019) previously posited an abnormal interaction within the salience network to describe misophonia (see Neacsiu et al. 2022 for a review), and our finding provides further evidence that the abnormal anterior insula connectivity in misophonia is specific to the salience network.

Further, the clusters to which the anterior insula were significantly connected as a function of misophonia severity overlap with areas associated with misophonia from previous literature, suggesting convergent mechanisms and compelling regions to explore further. We caution over‐interpretation of specific regions, since clusters reported herein are corrected for analysis at the cluster—not voxel—level, making interpretation of the cluster as a whole most appropriate rather than the voxels comprising it. However, it is worth consideration that clusters connecting to the anterior insula reported in this manuscript overlap anatomical regions that have been referenced in previous work. For instance, recent neuroimaging studies have likewise demonstrated anterior insula connectivity in misophonia to the supplementary motor cortex and precentral gyrus (Kumar et al. 2021; Hansen et al. 2022), which some researchers explain with a framework of misophonia that focuses on orofacial action perception and mimicry behaviors (Kumar et al. 2021; Berger et al. 2024). Alternatively, initial evidence suggests at least 25% of individuals with misophonia additionally experience misokinesia (Jaswal et al. 2021)—an aversion to movements—which may contribute to these underlying differences in motor cortex connectivity. Notably, as evident in Figures 3 and 5 and detailed in Tables S1–S3 and S5, the superior/medial portion of motor cortex shows peak connectivity in our study, which is known for representing movement of hands and fingers and was previously reported in Hansen et al. (2022). This finding, in combination with significant connectivity extending into the postcentral gyrus, a somatosensory region, further emphasizes a need for nonorofacial (e.g., finger) and nonmotor explanations when theorizing about mechanisms of misophonia (Hansen et al. 2021, 2022).

Aside from somatomotor regions, other areas found in this study to be significantly connected to the anterior insula as a function of misophonia are noteworthy. We found significant clusters of connectivity overlapping the planum temporale, which is a higher‐order auditory processing region in the superior temporal gyrus known for analyzing diverse types of complex sounds (Griffiths and Warren 2002). While connectivity of the planum temporale to motor cortex has been investigated in misophonia (Kumar et al. 2021; Hansen et al. 2022), to the best of our knowledge no study to date has reported on connectivity between the planum temporale and anterior insula in misophonia. Since these two regions involve functions especially relevant to the disorder, this strong association provides a compelling mechanism to explore more deeply, perhaps with neuromodulation techniques that can infer causality such as transcranial magnetic stimulation (see Neacsiu et al. 2022). Also, the present study found significant clusters of connectivity overlapping the posterior cingulate cortex, a region with diverse functions of higher‐order cognition that integrates prior experience with information from sensory systems (Foster and Koslov 2025). This region was additionally found to be significantly connected to the anterior insula in the earliest neuroimaging research on misophonia (Kumar et al. 2017). Taken together, our results support previous suggestions (Neacsiu et al. 2022) that the neurobiological mechanism of misophonia extends beyond a mere acoustic sensitivity, instead involving abnormal integration of stimulus salience with higher order auditory and cognitive processes.

### 4.2 The Insular Connectivity Profile of Misophonia Is Independent From Anxiety, Depression, or Autistic Traits

Second, we investigated how this insular connectivity profile of misophonia compared to other potentially related disorders. Using additional self‐report questionnaires available in the WAND dataset, we repeated our seed‐based connectivity analyses from the anterior insula as a function of anxiety, depression, and autism symptoms. While anxiety and depression each revealed significant insular connectivity profiles, the resultant clusters did not meaningfully overlap the clusters observed in misophonia. Further, the anterior insula was not significantly connected to any voxels as a function of autistic traits. To verify that our misophonia results were not unintentionally impacted by the presence of these disorders, we completed one final seed‐based connectivity analysis of misophonia severity with all available clinical metrics (anxiety, depression, and autism) and demographics (age and sex) treated as nuisance covariates of no interest. Importantly, the anterior insula remained significantly connected to the clusters observed in Figure 3. These results provide evidence that misophonia is a disorder that is neurally distinct from anxiety, depression, and autism.

We also presented behavioral correlations between misophonia severity and each of the assessment scores. In contrast to many previous studies, we found no significant behavioral correlations in our sample. We see a few possible explanations: First, similar to the null clinical findings and rationale of Grossini et al. (2022), perhaps our sample was too mild in symptoms to find an effect. It is possible that less extreme cases of misophonia are less likely to show comorbid psychopathology, whereas more extreme cases might. Second, perhaps the variability in assessment scales used across studies—both to assess misophonia, and to measure other clinical disorders—is contributing to the lack of cohesive results across groups. Assessment scales may be prefaced by disparate or imprecise instructions, prompting participants to respond differently to the same item if approached from an alternate mindset (e.g., “I prefer to do things with others” might not be endorsed by a misophonic individual after finishing a triggering experiment or answering misophonia‐related questionnaires about triggering sounds produced by others, but could be a trait they identify with if misophonia is not mentioned.) It is also possible that certain questions or scales better capture the similarity between misophonia and other clinical disorders than others. This may have been especially true here for the measurement of autistic traits: the AQ‐S covers difficulties with social skills, a preference for routine, attention‐switching difficulties, difficulties in imagination, and a fascination with numbers/patterns; noticeably absent are questions probing sensory processing dysfunction, which is a common experience in the autistic population (Tomchek and Dunn 2007; DuBois et al. 2017; Ben‐Sasson et al. 2019; Schauder and Bennetto 2016). In fact, investigating auditory hyperresponsivity and sound intolerance through the lens of misophonia has recently become a particular topic of scientific interest (Williams et al. 2021; Dwyer et al. 2025). While other research has shown higher AQ scores across these five traits in adults with misophonia (Rinaldi et al. 2023), it has done so using a different misophonia assessment (Sussex Misophonia Scale as opposed to MQ), further demonstrating that results may be largely dependent on the assessment scale used, and potentially the amount of overlapping information at the item level. Lastly, in contrast to these possibilities, perhaps these null findings add to the growing consensus of misophonia research that misophonia is a unique disorder (Erfanian et al. 2019; Siepsiak et al. 2020, 2022; Schröder et al. 2013; Taylor 2017) and is not systematically associated with other clinical assessments.

### 4.3 Misophonia Is Better Assessed as a Spectrum Than a Binary

In addition to these main findings, our results revealed supplementary insight worth discussing. While our primary analyses incorporated misophonia severity as a continuous measure (MQ total score), we also split the sample into “clinical misophonia” versus “subclinical misophonia” using the recommended clinical cutoff score of 30 (Möllmann et al. 2023) to better align with previous functional connectivity research in misophonia which employed binary groupings. Importantly, classifying participants in this way did not reveal any significant results like treating misophonia as a continuous variable did. This finding aligns with statistical expectations that splitting a continuous variable into a binary variable artificially reduces variability and thus statistical power. Additionally, it reinforces the idea that misophonia exists on a spectrum in the general population, and that studying the disorder as such can be more informative than treating it as a condition that is either present or absent (Norris et al. 2022).

Moreover, results from our linear aggression model built in the Oklahoma sample further support this position. As depicted in Figure 1, there was substantial overlap in MQ total scores between individuals self‐reporting clinical levels of misophonia on the MQ Severity Scale versus those self‐reporting nonclinical levels. While MQ total scores were significantly correlated with MQ Severity Scale responses (Figure S1), it is clear that binarizing the sample according to a single self‐report question is prone to noise: some individuals may perceive the impact of their misophonia to be high despite symptoms that are less prominent, whereas others might discount their functional disability but respond to individual questions with obvious symptom experience. With this in mind, we encourage future studies of misophonia to use continuous rather than categorical analytic approaches to better reflect the variability of misophonia symptoms in the real world; results may be missing useful information if only the latter is used.

### 4.4 Open‐Access Data Can Be Leveraged to Study Misophonia

Finally, this work demonstrates the ability to employ large, open‐access neuroimaging databases to study misophonia. Neuroimaging studies are expensive and time‐consuming; in lieu of individual labs collecting their own data, collaboration and data‐sharing can make large‐scale neuroimaging research more feasible (Lifshitz et al. 2012). By leveraging existing behavioral misophonia data containing pertinent assessments also used in the open‐access dataset, we were able to extrapolate misophonia in the open‐access dataset. While this strategy inherently limits the types of questions researchers can ask, it can still provide meaningful insight and participant diversity not previously attainable with an in‐house project.

### 4.5 Limitations and Future Directions

Accordingly, however, this study has a few notable limitations. Primarily, the participants with neuroimaging data used in this study were not directly assessed for misophonia; misophonia scores were predicted using a linear regression model built out of sample. While we find it encouraging that misophonia symptoms can be extrapolated in this way, there is no guarantee that participants in the sample would have self‐reported misophonia scores consistent with the model prediction. Similarly, the sample was of “healthy” adults from the general population in the UK and were thus not specifically recruited for misophonia complaints or assessed for misophonia by a trained clinician. Moreover, while the WAND sample contains a wealth of neuroimaging and behavioral data, certain audiological conditions related to misophonia (e.g., tinnitus, hyperacusis) were not assessed. Thus, we cannot rule out these confounds as contributing factors to the observed connectivity patterns. Our results indicate that relationships between functional connectivity and misophonia severity can still be observed in the general population, but, nevertheless, future work should seek to specifically recruit individuals with more severe misophonia and conduct more rigorous audiological screening. Further, it is worth pointing out that this study is based on resting‐state connectivity, so no misophonic stimuli (triggering or otherwise) were presented to participants. Follow‐up work may seek to test these hypotheses with task‐based fMRI, incorporating presentation of trigger sounds and analyzing activation or connectivity of the insula during sound presentation. Alternatively, since individual differences abound in which specific stimuli are most bothersome for individuals with misophonia, presenting the same “trigger” stimulus for every participant in a study is not guaranteed to elicit a maximal misophonic response for everyone; thus, future studies could employ internal imagination of triggering scenarios, the process of which has been shown in the memory literature to share core underlying neural processes as experiencing the same situations externally (Ferris et al. 2024).

Lastly, the present work is reliant on misophonia and clinical assessment scales present in the datasets and is thus restricted by the questions asked. Misophonia severity was solely assessed with the MQ (Wu et al. 2014), a popular metric released in 2014. While experts offered an updated definition of misophonia in 2022, there is still no consensus on a single assessment scale to use for misophonia; aspects of the consensus definition were incorporated into the Duke‐Vanderbilt Misophonia Screening Questionnaire (Williams et al. 2022) developed around the same time, but no scales to the best of our knowledge have specifically been designed based on the consensus definition. Given that misophonic individuals may respond perplexingly differently on different scales (see Hansen et al. 2024), and as demonstrated in this study, the single subjective measure of impairment used to threshold clinical severity on the MQ may not correspond perfectly to the total symptom severity score threshold, suggesting reduced internal validity for some questions, we recommend that multiple assessments should be collected and incorporated in research for a clearer picture of misophonic experiences, if possible. Thus, future work should replicate these findings using newer validated scales as they become available to better keep up with the rapidly changing literature.

## 5 Conclusion

Overall, our results provide neural evidence that misophonia exists on a spectrum, with observable differences in a large sample from the general population in which misophonia experiences vary. Specifically, these differences reveal a characteristic neural signature of misophonia marked by atypical salience insular connectivity: as misophonic symptom severity increases, functional connectivity increases between the anterior insula of the salience network and clusters overlapping regions such as the planum temporale and precentral gyrus. Further, this neural signature is only evident as a function of misophonia symptoms and not symptoms of anxiety, depression, or autism. These results underline the importance of the salience‐network anterior insula in understanding misophonic aversion and provide tentative evidence of neurological differences between misophonia and anxiety, depression, and autism. Future work can expand these findings to investigate the similarity between misophonia and other potentially related disorders (e.g., Obsessive–Compulsive Disorder, Sensory Processing Disorder). Additionally, given the multimodal nature of the WAND dataset, future work can utilize other neuroimaging methods to get a clearer picture of how misophonia presents in the brain.

## Funding

This work was supported by Réseau en Bio‐Imagerie du Quebec, Fonds de Recherche du Québec—Santé, Killam Trusts, and seed funding from the OU Data Institute for Societal Challenges.

## Ethics Statement

All methods and analyses presented in this paper were approved by the respective institutional ethics boards (Oklahoma dataset: University of Oklahoma Institutional Review Board; WAND dataset: Cardiff University School of Psychology Research Ethics Committee; Neuroimaging analyses: McGill Faculty of Medicine and Health Sciences Institutional Review Board). This article reports human subjects. Recruitment meets scientific requirements & HBMs expectation of inclusivity.

## Conflicts of Interest

The authors declare no conflicts of interest.

## Supporting information


**Data S1:** hbm70468‐sup‐0001‐Supinfo.docx.

## Data Availability

The neuroimaging data used in this paper are available for download by the authors of the WAND (see McNabb et al. 2025 for details). The Oklahoma dataset will be made readily available upon reasonable request.
