# Supplementary material for: Selective Disruption of Salience‐Network Anterior Insula Connectivity in Misophonia: A Disorder‐Specific Neural Signature
Source: Hum Brain Mapp. 2026 Feb 12;47(3):e70468. doi: 10.1002/hbm.70468 (PMC12895373; doi:10.1002/hbm.70468)
Supplement: Supplementary file 1 — Data S1: hbm70468‐sup‐0001‐Supinfo.docx. [file HBM-47-e70468-s001.docx]

# Supplement

## Aim 1


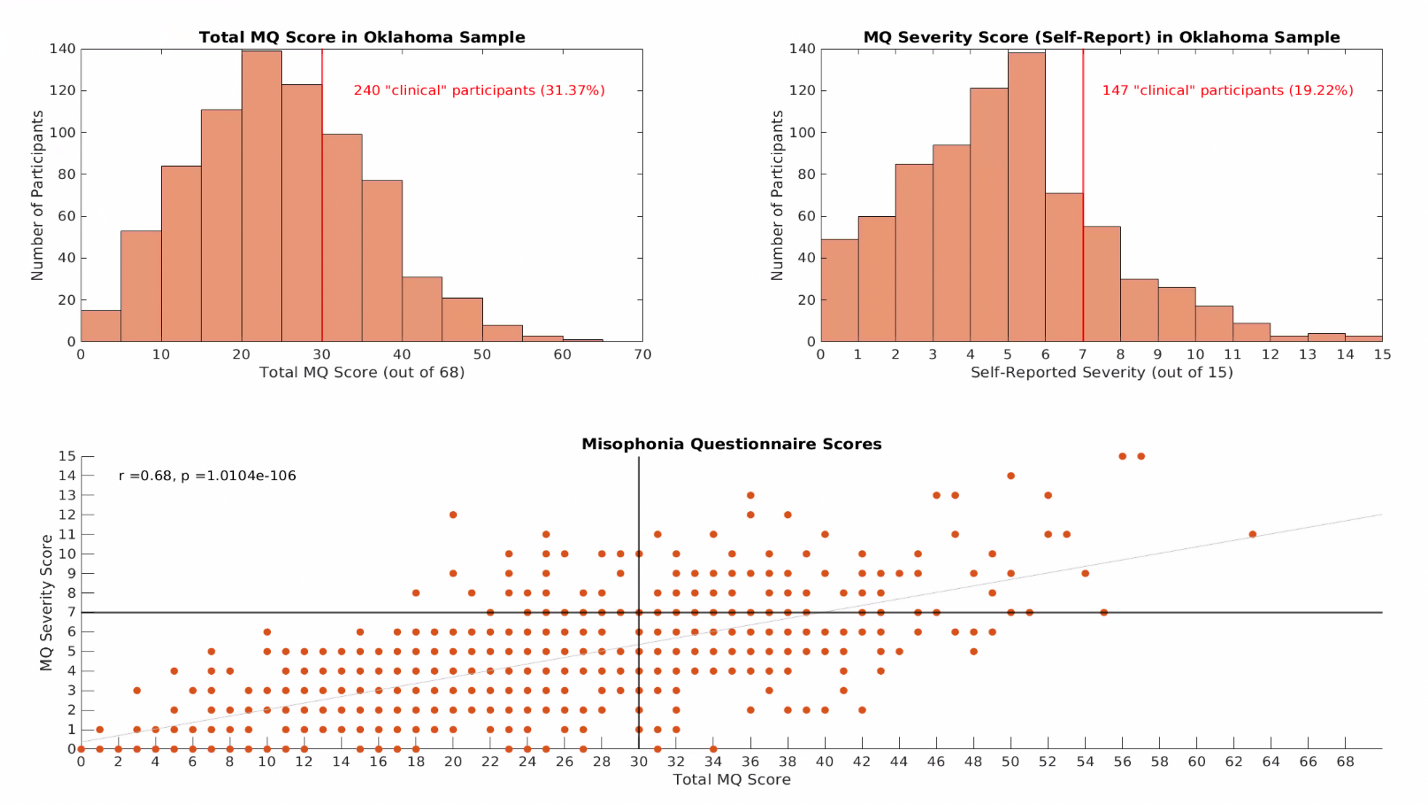


**Figure S1.** Top: Distribution of responses to the Misophonia Questionnaire (MQ) in the Oklahoma sample, with suggested clinical cutoffs depicted by red lines. Bottom: Correlation between total MQ score and self-reported severity score.

## Aim 2

**Table S1**. Peak voxel locations for significant clusters found connected to the anterior insula (defined by the default CONN parcellation) as a function of misophonia severity.

| **Region Name** | **Extent** | **Peak *t*** | **x** | **y** | **z** |
| --- | --- | --- | --- | --- | --- |
| *Cluster 1* | 1603 |  |  |  |  |
| Right Insular Cortex |  | 4.1257 | +28 | +10 | +10 |
| Right Central Opercular Cortex |  | 3.4969 | +48 | -12 | +10 |
| Right Planum Temporale |  | 3.4813 | +48 | -30 | +16 |
| Right Precentral Gyrus |  | 2.7605 | +54 | +4 | +16 |
| Right Temporal Pole |  | 2.7431 | +40 | +10 | -22 |
| *Cluster 2* | 2403 |  |  |  |  |
| Right Precentral Gyrus |  | 3.9596 | +20 | -14 | +62 |
| Right Supplementary Motor Cortex |  | 3.7976 | +2 | -6 | +54 |
| Left Supplementary Motor Cortex |  | 3.5461 | -8 | +2 | +48 |
| Right Superior Frontal Gyrus |  | 3.2699 | +14 | -6 | +72 |
| Left Superior Frontal Gyrus |  | 3.1435 | -14 | -8 | +68 |
| Left Posterior Cingulate Gyrus |  | 2.7907 | -8 | -16 | +42 |

Locations are labeled using the Harvard-Oxford cortical atlas. Coordinates are in MNI space.

**Table S2.** Peak voxel locations for significant clusters found connected to the Schaefer 100 Atlas LH_SalVenAttn Insula ROIs as a function of misophonia severity.

| **Region Name** | **Extent** | **Peak *t*** | **x** | **y** | **z** |
| --- | --- | --- | --- | --- | --- |
| *Cluster 1* | 1116 |  |  |  |  |
| Right Precentral Gyrus |  | 3.5234 | +40 | -10 | +56 |
| Right Supplementary Motor Cortex |  | 3.1758 | +4 | -6 | +54 |
| Right Postcentral Gyrus |  | 3.0155 | +50 | -12 | +50 |
| Left Supplementary Motor Cortex |  | 2.8669 | -6 | +2 | +46 |

Locations are labeled using the Harvard-Oxford cortical atlas. Coordinates are in MNI space.

**Table S3.** Peak voxel locations for significant clusters found connected to the left anterior insula and right anterior insula (defined by the default CONN parcellation) as a function of misophonia severity.

| **Seed** | **Region Name** | **Extent** | **Peak *t*** | **x** | **y** | **z** |
| --- | --- | --- | --- | --- | --- | --- |
| **Left** Anterior Insula | *Cluster 1* | 1158 |  |  |  |  |
|  | Right Precentral Gyrus |  | 4.0265 | +22 | -12 | +62 |
|  | Right Supplementary Motor Cortex |  | 3.2443 | +2 | -6 | +54 |
|  | Left Supplementary Motor Cortex |  | 2.9854 | -8 | -10 | +62 |
|  | Right Superior Frontal Gyrus |  | 2.7453 | +12 | -8 | +74 |
|  | Left Superior Frontal Gyrus |  | 2.5200 | -16 | -10 | +72 |
|  | Right Postcentral Gyrus |  | 2.4860 | +52 | -12 | +52 |
| **Right** Anterior Insula | *Cluster 1* | 1464 |  |  |  |  |
|  | Left Frontal Operculum Cortex |  | 3.7270 | -36 | +12 | +16 |
|  | Left Central Opercular Cortex |  | 3.6565 | -44 | -12 | +18 |
|  | Left Precentral Gyrus |  | 3.4012 | -46 | -2 | +20 |
|  | Left Insular Cortex |  | 3.3050 | -28 | +2 | +14 |
|  | Left Frontal Orbital Cortex |  | 3.0928 | -18 | +8 | -14 |
|  | Left Heschl's Gyrus |  | 3.0698 | -56 | -16 | +6 |
|  | Left Temporal Pole |  | 2.8177 | -26 | +4 | -18 |
|  | Left Anterior Superior Temporal Gyrus |  | 2.7897 | -52 | -14 | -2 |
|  | Left Planum Polare |  | 2.6102 | -50 | +2 | -6 |
|  | Left Posterior Superior Temporal Gyrus |  | 2.4533 | -48 | -26 | 0 |
|  | *Cluster 2* | 1639 |  |  |  |  |
|  | Right Insular Cortex |  | 4.7366 | +36 | 0 | +6 |
|  | Right Central Opercular Cortex |  | 3.4459 | +48 | -12 | +12 |
|  | Right Inferior Frontal Gyrus (pars opercularis) |  | 3.3269 | +52 | +16 | +2 |
|  | Right Temporal Pole |  | 3.1010 | +40 | +10 | -22 |
|  | Right Inferior Frontal Gyrus (pars triangularis) |  | 2.8453 | +50 | +30 | +4 |
|  | Right Frontal Pole |  | 2.7693 | +52 | +38 | +10 |
|  | Right Posterior Superior Temporal Gyrus |  | 2.6010 | +48 | -12 | -10 |
|  | *Cluster 3* | 3238 |  |  |  |  |
|  | Right Postcentral Gyrus |  | 4.0243 | +20 | -34 | +58 |
|  | Right Precuneus Cortex |  | 3.7531 | +14 | -40 | +52 |
|  | Left Supplementary Motor Cortex |  | 3.7398 | -2 | -6 | +56 |
|  | Right Supplementary Motor Cortex |  | 3.6504 | +6 | +6 | +54 |
|  | Right Precentral Gyrus |  | 3.5721 | +18 | -14 | +62 |
|  | Left Postcentral Gyrus |  | 3.5609 | -18 | -40 | +56 |
|  | Right Superior Frontal Gyrus |  | 3.5600 | +12 | -6 | +64 |
|  | Left Precentral Gyrus |  | 3.5102 | -10 | -20 | +44 |
|  | Left Superior Frontal Gyrus |  | 3.4707 | -16 | -8 | +68 |

Locations are labeled using the Harvard-Oxford cortical atlas. Coordinates are in MNI space.

**Table S4.** Clinical breakdown of the WAND sample, separated by MQ total scores ≥30 (misophonia “patients”) versus MQ total scores <30 (“controls”). Caseness is assigned using terminology of the respective publications cited in text.

| **Clinical Measure** | **Misophonia "Patients" (N = 31)** | **"Controls"**  **(N = 131)** | **Full Sample**  **(N = 162)** |
| --- | --- | --- | --- |
| ***Predicted MQ total score*** |  |  |  |
| M (SD) | 33.9 (4.2) | 21.9 (6.6) | 24.2 (6.6) |
| range | 30.2-45.1 | 8.9-29.7 | 8.9-45.1 |
| ***Anxiety (HADs)*** |  |  |  |
| "clinical" cases, N (%) | 3 (9.7) | 18 (13.7) | 21 (13.0) |
| "borderline" cases, N (%) | 3 (9.7) | 17 (13.0) | 20 (12.3) |
| ***Depression (HADs)*** |  |  |  |
| "clinical" cases, N (%) | 1 (3.2) | 2 (1.5) | 3 (1.9) |
| "borderline" cases, N (%) | 0 (0.0) | 7 (5.3) | 7 (4.3) |
| ***Autism (AQ-S)*** |  |  |  |
| presence of autistic traits, N (%) | 6 (19.4) | 24 (18.3) | 30 (18.5) |

MQ = Misophonia Questionnaire. HADs = Hospital Anxiety and Depression Scale. AQ-S = Autism-Spectrum Quotient Short.

**Table S5.** Peak voxel locations for significant clusters found connected to the anterior insula (defined by the default CONN parcellation) as a function of misophonia severity, after controlling for all other clinical and demographic covariates.

| **Region Name** | **Extent** | **Peak *t*** | **x** | **y** | **z** |
| --- | --- | --- | --- | --- | --- |
| *Cluster 1* | 1537 |  |  |  |  |
| Right Insular Cortex |  | 4.1395 | +28 | +10 | +10 |
| Right Central Opercular Cortex |  | 3.5880 | +48 | -12 | +10 |
| Right Planum Temporale |  | 3.4972 | +48 | -30 | +14 |
| Right Parietal Operculum Cortex |  | 3.4361 | +50 | -30 | +20 |
| Right Precentral Gyrus |  | 2.7980 | +54 | +4 | +16 |
| *Cluster 2* | 3195 |  |  |  |  |
| Right Precentral Gyrus |  | 4.0136 | +22 | -12 | +64 |
| Right Supplementary Motor Cortex |  | 3.8118 | +2 | -6 | +54 |
| Left Supplementary Motor Cortex |  | 3.5486 | -8 | +2 | +48 |
| Right Postcentral Gyrus |  | 3.4484 | +20 | -36 | +60 |
| Right Superior Frontal Gyrus |  | 3.1406 | +14 | -6 | +72 |
| Left Superior Frontal Gyrus |  | 2.9079 | -22 | -2 | +64 |
| Left Posterior Cingulate Gyrus |  | 2.6439 | -8 | -16 | +42 |

Locations are labeled using the Harvard-Oxford cortical atlas. Coordinates are in MNI space.
